# Ectopic expression of miR‐944 impairs colorectal cancer cell proliferation and invasion by targeting GATA binding protein 6

**DOI:** 10.1111/jcmm.14245

**Published:** 2019-03-15

**Authors:** Jing‐Tong Tang, Jinbo Zhao, Weiwei Sheng, Jian‐Ping Zhou, Qi Dong, Ming Dong

**Affiliations:** ^1^ Department of Gastrointestinal Surgery & Hernia and Abdominal Wall Surgery The First Hospital, China Medical University Shenyang Liaoning China; ^2^ Department of General Surgery The People's Hospital of China Medical University Shenyang China

**Keywords:** colorectal cancer, GATA6, invasion, migration, miR‐944, proliferation

## Abstract

miR‐944 is a microRNA that has been reported to play different important roles in the progression of cancer. Colorectal cancer (CRC) is a common cancer worldwide. A recent study has confirmed that miR‐944 plays a tumour suppressive role in CRC. However, biological functions and the mechanism of miR‐944 in CRC are poorly understood. Real‐time reverse transcription polymerase chain reaction of 100 CRC tissues showed that miR‐944 expression is frequently downregulated and is negatively associated with the T is the primary tumor, N is the lymph node, and M is the distant metastasis (TNM) stage (*P* = 0.009), depth of invasion (*P* = 0.001), and lymph node status (*P* = 0.002). Overexpression of mir‐944 significantly impaired the functions of proliferation, migration and invasion in CRC cells, while these functions increased in knockdown experiments. GATA binding protein 6 (GATA6) knockdown can reverse the CRC cells functions induced by miR‐944 inhibitor. Mechanistically, a Dual‐Luciferase Reporter Assay showed that miR‐944 is structurally combined with GATA6 and interacts with downstream proteins (CRT and p‐AKT) in CRC cells. In conclusion, these findings indicated that miR‐944 may be a tumour suppressor and could likely be used as a prognostic predictor and novel therapeutic target for CRC.

## 1 INTRODUCTION

As the spectrum of diseases changes, malignant tumours have become a common cause of death. Colorectal cancer (CRC) is a common malignant tumour 1, 2; the incidence rate is ranked fourth, which is still increasing worldwide; and the mortality rate of CRC has been reported to be 9.2% every year.^3^ Although advances have been made in clinical and mechanistic research, the 5‐year survival rate remains dismal. Therefore, further study of the underlying mechanism of CRC remains necessary to determine a more effective diagnosis and treatment of CRC.

Many studies have reported that miRNAs play an important role in the formation and development of tumours.^4^, ^5^ First described by Lee et  al in 1993,^6^ miRNAs are a series of short, conserved, noncoding RNAs that can act as tumour promotors or tumour inhibitors in the human body by affecting the expression of target proteins. Mechanistically, miRNAs can imperfectly combine with the 3′‐untranslated region (3′‐UTR) of target mRNAs to inhibit the translation of mRNA.^7^, ^8^ miRNAs regulate key cell functions, such as proliferation, invasion, migration, apoptosis, and immunity.^9^, ^10^

Previous studies have shown that microRNA (miR‐944) expression is abnormal and plays either tumour suppressive or oncogenic roles in human malignancies. In breast cancer, Ali Flores‐Pérez et  al^11^ found that miR‐944 acts as an inhibitor by targeting SIAH1 and PTP4A1. However, in cervical cancer studies, miR‐944 plays an oncogenic role by targeting HWCW2 and S100PBP.^12^ A recent study showed that the expression level of miR‐944 in tumour tissues is less than that in adjacent non‐tumour tissues in CRC.^13^ Another study showed that the expression level of miR‐944 was frequently low in recurrent CRC patients.^14^ Dysregulation of GATA transcription factor 6 (GATA6) has been reported to affect the initiation and progression of tumours. In breast cancer, GATA6 can stimulate EMT by inhibiting the E‐cadherin protein.^15^ Strong expression of GATA6 is associated with liver metastasis and poor patient survival in CRC.^16^ However, the role of miR‐944 and its correlation with GATA6 in CRC have not been reported clearly. Therefore, we evaluated the expression and potential mechanism of signalling pathways in CRC.

## 2 MATERIALS AND METHODS

### 2.1 Human tissue specimens and cell lines

A total of 100 pairs of fresh colorectal tissue and matched adjacent non‐tumour colorectal tissue were all collected from CRC patients who underwent colorectal surgery in the Department of Gastrointestinal Surgery at the First Affiliated Hospital of China Medical University and were all diagnosed with CRC histologically. The patients included 68 males and 32 females (median age of 61.78 years; range of 41‐85 years). None of these patients were subjected to adjuvant therapy before surgery. The specimens were immediately stored at −80°C or embedded in paraffin after surgical resection. This study was approved by the Ethics Committee of China Medical University.

Four CRC cell lines (HT‐29, Human colon cancer cells‐116 (HCT116), SW480, and SW620) and the human embryonic kidney 293 cell line were purchased from the Cell Bank at the Chinese Academy of Sciences (Shanghai, China). human embryonic intestinal mucosa cells (CCC‐HIE‐2) was obtained from the American Type Culture Collection (ATTC; Manassas, VA, USA). Four CRC cell lines and CCC‐HIE‐2 cells were cultured in RPMI‐1640 (BI, Shanghai, China) with 10% fetal bovine serum (FBS; Hyclone, Logan, UT)and 100 U/mL penicillin‐streptomycin, and human 293 cells were cultured in DMEM (BI) with 10% FBS and 100 U/mL penicillin‐streptomycin. The cells were all cultured in a humidified incubator with 5% CO_2_ at 37°C.

### 2.2 RNA isolation and quantitative real‐time PCR

All RNA was extracted from the CRC cell lines and colorectal tissue stored at −80°C by TRIzol reagent (Takara Bio, Otsu, Japan) following the manufacturer's instructions. Reverse transcription reactions and real‐time PCR reactions were performed by a Hairpin‐it microRNA and U6 snRNA Normalization RT‐PCR Quantitation Kit (GenePharma, Shanghai, China). The expression of miR‐944 was measured by a Light Cycler kit (Takara Bio) system according to the manufacturer's thermocycling conditions as follows: 95°C for 3 minutes, followed by 45 cycles at 95°C for 12 seconds and 62°C for 45 seconds. Here, U6 snRNA was used as an internal control. Fold changes (2^−ΔΔCt^) were used to analyse the relative expression of miR‐944. Each experiment was replicated three times.

### 2.3 Western blot analysis and co‐immunoprecipitation

Total protein was extracted using Radio‐Immunoprecipitation Assay lysis buffer containing 1% phenylindole sulfonylfluoride (PMSF) from two infected CRC cell lines (HCT116 and SW480). Harvested cell proteins were added to a 10% sodium dodecyl sulfate‐polyacrylamide gel and transferred onto polyvinylidene fluoride membranes (Millipore, Bedford, MA). The membranes were incubated with rabbit anti‐GATA6 (Abcam, Cambridge, UK), mouse anti‐CRT (Abcam), and mouse anti‐p‐AKT (Proteintech, Chicago, IL) primary antibodies at a dilution of 1:1000 and with a mouse anti‐GAPDH (Proteintech) primary antibody at a dilution of 1:3000 overnight at 4°C. The secondary antibodies were incubated with the membranes for 2 hours on the following day. The protein bands were analysed using an ECL detection kit (Thermo Fisher Scientific, Rockford, IL).

Co‐immunoprecipitation was performed with SW480 and HCT‐116 cell lines. After 48 hours, we collected the cells and added RIPA lysis buffer (composed of 20 mmol/L Tris/HCl (pH 7.4), 1.0% NP‐40, 150 mmol/L NaCl, 1 mmol/L ethylenediaminetetraacetic acid, 10 μg/mL leupeptin, and 50 μg/mL PMSF). Supernatants containing proteins were then collected and incubated with anti‐AKT (Proteintech) antibodies for 1 hour, followed by incubation with magnetic beads at 4°C for 18 hours. The samples were then subjected to SDS‐PAGE for separation and detection. Co‐precipitation was used to detect the CRT (Abcam) and AKT (Proteintech) antibodies, and endogenous coprecipitation was used to detect CRT protein bands. The experiment was repeated three times.

### 2.4 Immunohistochemistry

Colorectal cancer tissues embedded in paraffin were cut into 4‐μm‐thick continuous sections. After being deparaffinized at 65°C for 2 hours, antigen repair was performed at a high pressure for 2.5 minutes, and then slides were cooled at room temperature for 1 hour. The sections were incubated with H_2_O_2_ (3%) for 30 minutes to block endogenous peroxidase activity. Ten percent normal goat serum was added for 30 minutes to reduce nonspecific binding, and the sections were incubated with rabbit monoclonal anti‐GATA6 (Abcam) overnight at 4°C. On the following day, after adding a secondary antibody for 20 minutes, the sections were incubated with streptavidin‐peroxidase reagent for 20 minutes. 3,3′‐Diaminobenzidine (DAB) was added for signal reactions. The stained sections were imaged at ×20 magnification.

Immunohistochemistry (IHC) scoring was performed as described previously 17. The staining range score was defined as 1 (0%‐25%), 2 (25%‐50%), 3 (50%‐75%), and 4 (75%‐100%) according to the positively colored range of the slides. Staining intensity scores were defined as 0 (negative), 1 (weak), 2 (moderate) and 3 (strong). Multiplication of the two scores was the final score ranging from 0 to 12. A final score ≥8 was defined as a positive expression.

### 2.5 Transfection

An miR‐944 mimic and its corresponding negative control (referred as mimics‐NC) and an miR‐944 inhibitor and its corresponding NC (referred as inhibitor‐NC) were all synthesized at GenePharma. GATA6 siRNA (referred to as GATA6‐siRNA) and control siRNA (referred to as siRNA‐control) were synthesized at HANBIO (Shanghai, China). Transfection was performed using Lipofectamine 3000 (Invitrogen, Carlsbad, CA) according to the manufacturer's instructions.

### 2.6 Cell proliferation and colony formation assays

HCT‐116 and SW480 cells infected for 48 hours were harvested, and 2 × 10 ^4^ cells in 100 μL of the culture medium were added into each well of 96‐well plates and incubated in a CO_2_ incubator. Cell numbers were analysed at 24, 48, 72 and 96 hours. After 15 μL 3‐(4,5‐dimethyl‐2‐thiazolyl)‐2,5‐diphenyl‐2‐H‐tetrazolium bromide, Thiazolyl Blue Tetrazolium Bromide (MTT) reagent (5 mg/mL) was added to each well and incubated for 4 hours, 150 µL dimethyl sulfoxide (DMSO) was added to each well, and the absorbance was measured with an ELISA 96‐well microtiter plate reader (Bio‐Rad 680, California, CA) at a wavelength of 490 nm. For colony formation assays, 1000 harvested cells/well were added into six‐well plates. The cells were incubated at 37°C for 14 days. The colonies were stained with cold methanol and 0.1% crystal violet (Sigma‐Aldrich) for 30 minutes, and the number of cells was counted in three random fields per chamber at ×20 magnification. Each experiment was performed three times independently.

### 2.7 Cell migration and invasion assay

HCT‐116 and SW480 cells were infected as described previously. The experiment was performed using 24‐well Transwell plates and specialized Transwell chambers. For the invasion assay, a membrane coagulated with Matrigel (BD Biosciences) was added into the upper chamber. Infected cells (1 × 10^5^ cells/well) were suspended in 300 µL serum‐free medium and then added into the upper compartment of the chamber. The lower chamber contained 600 µL RPMI‐1640 and 10% FBS. After 24 hours of incubation, the cells that migrated through the Transwell membrane were stained with cold methanol and dyed with 0.1% crystal violet (Sigma‐Aldrich) for 30 minutes, and then the cells were counted in four random fields at 200× magnification. Each experiment was performed three times independently.

### 2.8 Dual‐luciferase reporter assay

For the luciferase assays, the 3′‐UTR of the GATA6 mRNA and mutant GATA6 mRNA were synthesized and inserted into pMIR‐REPORT Luciferase (OBIO, Shanghai, China), and the wild‐type and mutant sequences at 637‐643 were 5′‐UUCUUCCAUGUAUACAAUAAUUU‐3′ and 5′‐UUCUUCCAUGUAUACCGCGUACA‐3′, respectively, and the wild‐type and mutant sequences at 753‐759 were 5′‐CUUAAUGGUACUUAAAAUAAUUU‐3′ and CUUAAUGGUACUUACCGCGUACA‐3′, respectively. Human 293 cells were cultured in 12‐well plates, and when they were nearly 50% cotransfected with the pMIR‐REPORT luciferase‐GATA6 (0.4 µg) or pMIR‐REPORT‐mt‐GATA6 (0.4 µg) plasmid, the pRL‐TK luciferase reporter (100 ng/well) and the miR‐944 mimic (25 nmol/L) or mimics‐NC (25 nmol/L) were transfected, using Lipofectamine 3000 (Invitrogen). Twenty‐four hours later, the cells were harvested, and luciferase activities were measured, using a Varioskan Flash System (Thermo Fisher Scientific). Each experiment was performed three times independently.

### 2.9 Statistical analysis

spss 17.0 software (Chicago, IL) was used for all statistical analyses. The consecutive data are shown as the mean ± SD. Protein expression of GATA6, CRT, p‐AKT, AKT and the results of the colony formation assay and the Dual‐Luciferase Reporter Assay were analysed by the paired sample *t* test. The relationship between miR‐944 and the expression of GATA6, the association between miR‐944 and clinical pathological parameters in CRC, and the differences in the cell proliferation, cell migration and cell invasion assays were analysed by Student's *t* test. The relationship between miR‐944 and GATA6 was analysed by Spearman's correlation test. The results were considered to be statistically significant at *P* < 0.05 in all experiments.

## 3 RESULTS

### 3.1 The decreased expression level of miR‐944 in CRC tissue is associated with CRC patient clinical pathology

**Figure 1:**
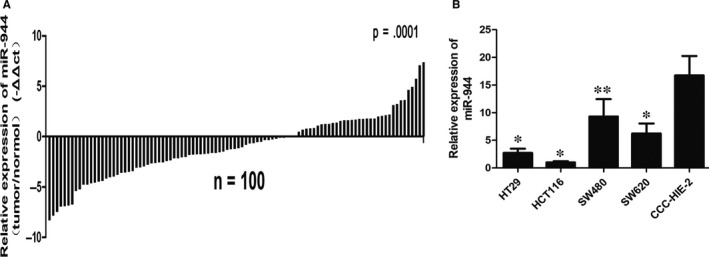
miR‐944 expression is frequently downregulated in human colorectal cancer (CRC). A, The relative expression of miR‐944 in 100 pairs of CRC tissues. miR‐944 expression was analysed by quantitative real time polymerase chain reaction (qRT‐PCR) (normalized to U6 snRNA). Data are shown as –ΔΔCT (tumour/normal). B, The relative expression of miR‐944 in four CRC cell lines and CCC‐HIE‐2 cells. Data are shown as 2^–ΔΔct^, n = 3, *P < 0.05 and **P < 0.01 by *t* test compared with CCC‐HIE‐2 cells

miR‐944 expression in 100 pairs of CRC tissues and corresponding adjacent non‐tumour tissues was examined by quantitative real time polymerase chain reaction (qRT‐PCR). Our data showed that 65 CRC tissues had low miR‐944 expression levels and 35 tissues had high expression levels (*P* = 0.001, Figure 1A). Then, we examined miR‐944 expression in four CRC cell lines and CCC‐HIE‐2 cells. We found that SW480 had the highest expression level of miR‐944 and that HCT‐116 had the lowest expression level of miR‐944 in four CRC cell lines. miR‐944 was significantly overexpressed in CCC‐HIE‐2 cells compared with four CRC cell lines (Figure 1B). Furthermore, we analysed the correlation between clinical pathology and the expression of miR‐944. We found that miR‐944 expression was negatively associated with the TNM stage (*P* = 0.009), depth of invasion (*P* = 0.001), and lymph node status (*P* = 0.002). However, there was no significant correlation with age, gender, tumour size, the degree of tumour differentiation and metastasis (Table 1).

**Table 1:** Relationship between clinicopathological characteristics and the expression of miR‐944 in CRC

Characteristics		N(100)	Expression of miR‐944		*P* value
			Mean	95% CI	
Age (y)					0.48
<60		48	20.35	6.76‐38.93	
≥60		52	13.64	5.71‐24.16	
Gender					0.66
Male		68	18.30	7.84‐33.10	
Female		32	13.80	2.87‐28.42	
Size (maximal diameter)					0.88
≥5 cm		41	16.01	4.93‐34.55	
<5 cm		59	17.46	7.29‐30.16	
Differentiation					0.72
Well, moderate		86	16.18	6.55‐27.37	
Poor		14	21.04	1.97‐48.00	
TNM stage					0.001**
I + II		64	25.44	12.63‐40.07	
III + IV		36	1.61	0.94‐2.32	
Tumour invasion					0.009**
T1 + T2 + T3		80	20.09	10.20‐32.12	
T4		20	3.96	1.52‐7.00	
Lymph node status					0.002**
Positive		34	1.61	0.94‐2.37	
Negative		66	24.72	13.20‐39.94	
Metastasis					0.32
M0		92	18.25	9.44‐29.63	
M1		8	0.90	0.37‐1.53	

The mean of the relative miR‐944 expression is showed as fold change and 95% confidence interval (CI).

CRC, colorectal cancer; miR‐944, microRNA‐944.

*P < 0.05, **P < 0.01.

### 3.2 miR‐944 inhibits CRC cell proliferation, migration, and invasion

**Figure 2:**
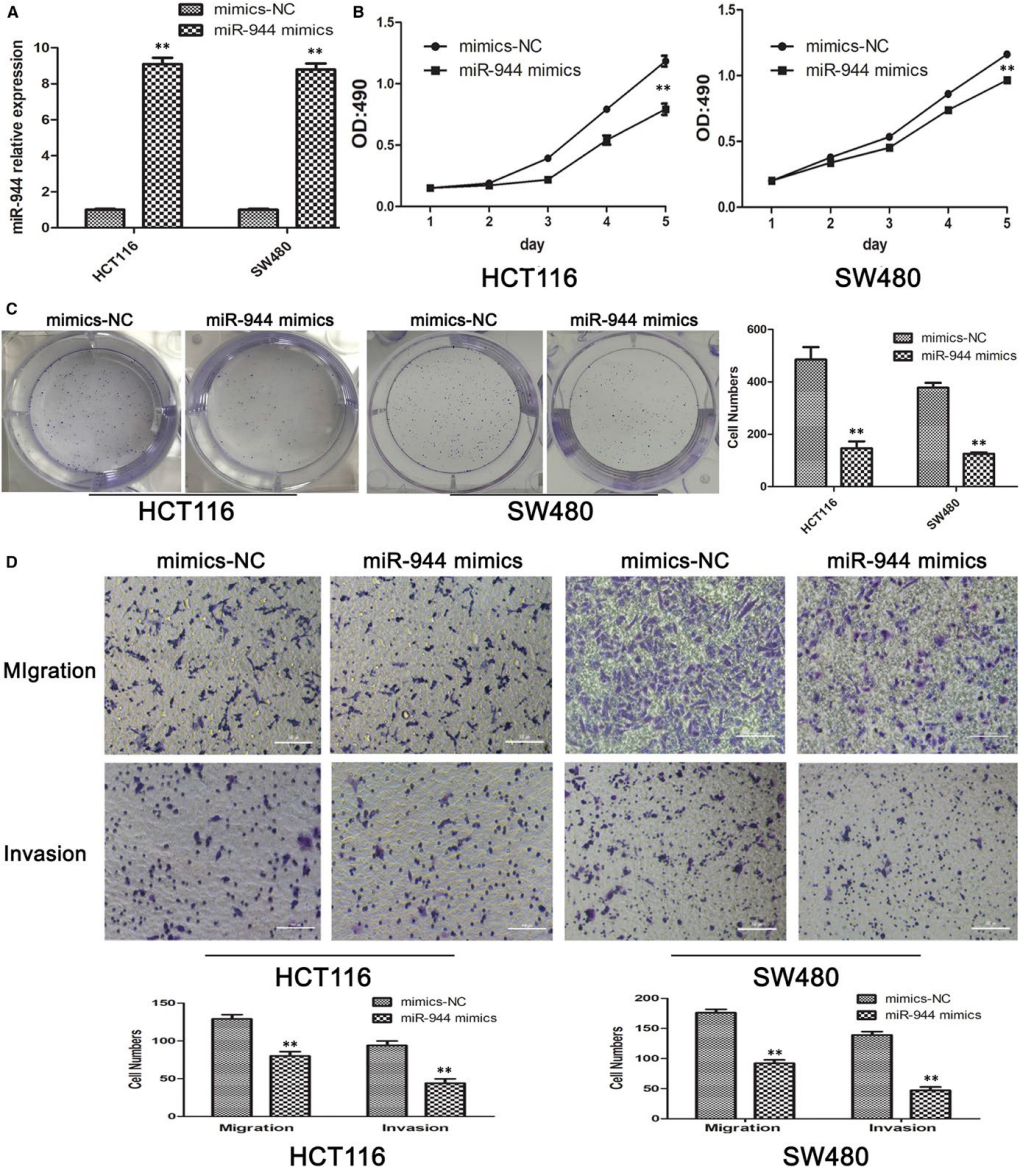
miR‐944 inhibits the proliferation, migration and invasion of Human colon cancer cells‐116 (HCT116) and SW480 cells. A, Overexpression of miR‐944 was confirmed by quantitative real time polymerase chain reaction (qRT‐PCR), n = 3, **P < 0.01 by *t* test. B, 3‐(4,5‐dimethyl‐2‐thiazolyl)‐2,5‐diphenyl‐2‐H‐tetrazolium bromide, Thiazolyl Blue Tetrazolium Bromide(MTT) assays showed that overexpression of miR‐944 inhibited cell proliferation, **P < 0.01. C, Colony formation assays showed that overexpression of miR‐944 inhibited cell proliferation, **P < 0.01. D, Migration and invasion assays showed that HCT116 and SW480 cells transfected with miR‐944 mimics inhibited cell migration and invasion, **P < 0.01

**Figure 3:**
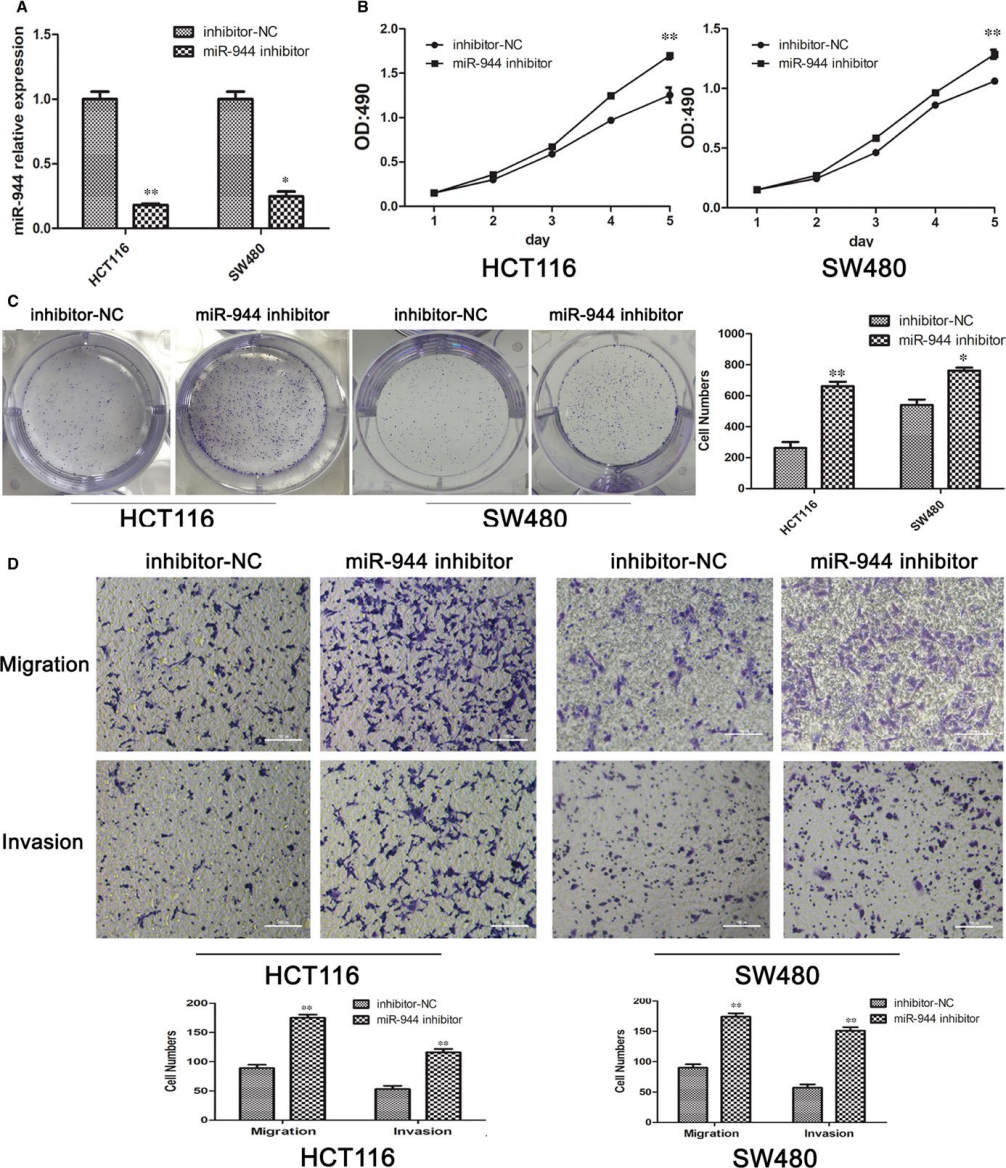
Knockdown of miR‐944 prompted the proliferation, migration and invasion of Human colon cancer cells‐116(HCT116) and SW480 cells. A, Downregulation of miR‐944 expression was confirmed by quantitative real time polymerase chain reaction (qRT‐PCR) and cell proliferation, *P < 0.05 and **P < 0.01. D, Migration and invasion assays of HCT116 and SW480 cells transfected with miR‐944 inhibitor, n = 3, *P < 0.05 and **P < 0.01 by *t* test. B, 3‐(4,5‐dimethyl‐2‐thiazolyl)‐2,5‐diphenyl‐2‐H‐tetrazolium bromide, Thiazolyl Blue Tetrazolium Bromide (MTT) assays showed that miR‐944 silencing promoted cell proliferation, **P < 0.01. C, Colony formation assays showed that miR‐944 silencing promoted cell migration and invasion, **P < 0.01

The tumour functions of proliferation, migration, and invasion are key factors that affect the TNM stage and patient survival. To determine the effect of miR‐944 on these functions, we used the highest and lowest miR‐944‐expressing CRC cell lines (SW480 and HCT116 cells, respectively) and transfected them with an miR‐944 mimic and its corresponding NC and an miR‐944 inhibitor and its corresponding NC. The transfection efficiency was analysed by qRT‐PCR (Figures 2A & 3A). However, miR‐944 overexpression significantly inhibited CRC cell proliferation, as indicated by the MTT (Figure 2B) and colony formation (Figure 2C) assays, and the Transwell assays showed that miR‐944 overexpression significantly reduced CRC cell migration and invasion compared with the NC (Figure 2D). In contrast, transfecting the cells with the miR‐944 inhibitor significantly decreased the expression level of miR‐944 and promoted CRC cell proliferation, migration and invasion (Figure 3).

### 3.3 miR‐944 targets the GATA6 gene in CRC cells

**Figure 4:**
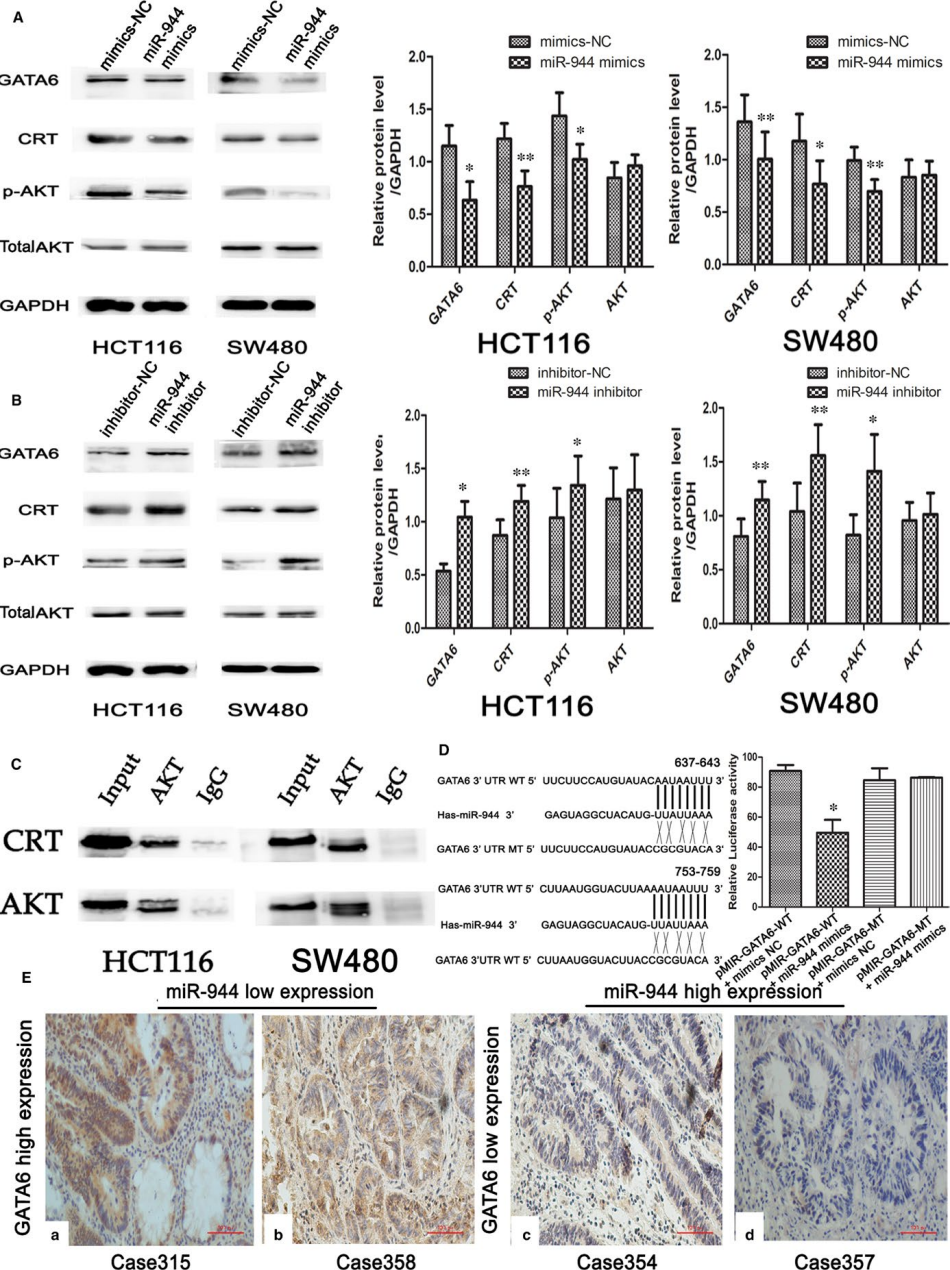
The association between miR‐944 expression and the GATA/CRT/p‐AKT signalling pathway in two colorectal cancer (CRC) cell lines (Human colon cancer cells‐116(HCT116) and SW480) and CRC tissues. A, Western blot analysis of GATA binding protein 6 (GATA6), CRT, p‐AKT, and AKT expression after transfecting cells with mimics‐NC and miR‐944 mimics. B, Western blot analysis of GATA6, CRT, p‐AKT, and AKT expression after transfecting cells with inhibitor‐NC and miR‐944 inhibitor. C, AKT co‐immunoprecipitated with CRT in HCT 116 and SW480 cells. The input and IgG lanes show the positive and negative controls, respectively. D, Analysis of the relative luciferase activities of the GATA6 WT 3ʹ‐untranslated region (3ʹ‐UTR) and GATA6 MUT 3ʹ‐UTR reporter vectors following transfection with miR‐944 mimics or mimics‐NC in HEK293 cells. E, The association between GATA6 and miR‐944 expression levels in CRC tissues. (a,b) A low expression level of GATA6 was observed in tissues with high expression levels of miR‐944. (c,d) A high expression level of GATA6 was observed in tissues with low expression levels of miR‐944

To explore the potential mechanism by which miR‐944 affects CRC cell function, we used two databases that predict the miRNA targets (microRNA and TargetScan) and found that GATA6 may be a potential target. We transfected miR‐944 mimics into SW480 and HCT116 cells, and the expression of GATA6 was downregulated (Figure 4A). Furthermore, the expression level of GATA6 increased in the miR‐944 knockdown experiment (Figure 4B). Moreover, we found that the cancer tissue with high expression levels of miR‐944 showed GATA6 protein expression negativity (14/16; Figure 4E c,d, Table S1). Low expression levels of miR‐944 showed GATA6 positivity (16/24; Figure 4E a,b, Table S1) in the IHC of 40 pairs of CRC patients collected recently (*P* = 0.001) (Table 2). In conclusion, we used GATA6 as the target of miR‐944.

**Table 2:** Expression association between miR‐944 and GATA6 (n = 40) in primary CRC

	Expression of miR‐944		*P* value
	Low expression (n = 24)	High expression (n = 16)	
GATA6			
Negative	8	14	0.001
Positive	16	2	

CRC, colorectal cancer; miR‐944, microRNA‐944.

To further confirm the combination, the GATA6 3′‐UTR‐binding sequence (wild type and mutant type), miR‐944 was inserted into the pmiR‐REPORT plasmid. Then, we cotransfected two pmiR‐REPORT plasmids (wild‐type and mutant‐type) with a mimics‐NC and an miR‐944 mimic into HEK293 cells. As shown in Figure 4D, we found that transfection of the miR‐944 mimics significantly reduced the luciferase activity compared with NC in cells infected with the wild‐type 3′‐UTR, while there was no significance in cells infected with the mutant‐type 3′‐UTR pmiR‐REPORT plasmid. In conclusion, these results showed that GATA6 is a direct target of miR‐944.

### 3.4 miR‐944 affects GATA6 and its downstream proteins in CRC cell lines

**Figure 5:**
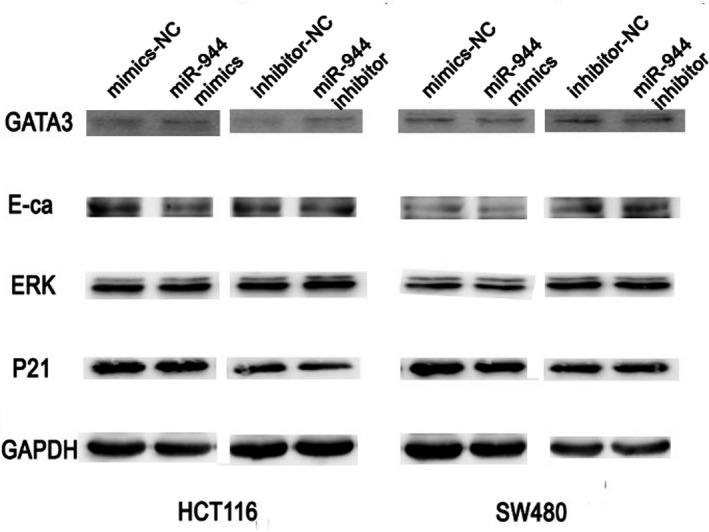
No differential protein expression levels were observed in the overexpression and knockdown experiments. The expression of GATA3, E‐ca, ERK and P21 was not affected by the overexpression or knockdown of miR‐944. E‐ca: E‐cadherin

GATA6 is a transcription factor that binds to the promoter of CRT. Our study also showed that miR‐944 mediated the downregulation of CRT protein expression by inhibiting GATA6 protein expression (Figure 4A,B). Immunoprecipitation showed that CRT has a close interaction with AKT (Figure 4C). The p‐AKT protein expression level was decreased in the miR‐944 mimic group compared with the corresponding NC group and was increased in the miR‐944 inhibitor group compared with the NC group in two CRC cell lines without changing the expression of total AKT proteins (Figure 4A,B). In this study, we also examined other proliferation‐, migration‐ and invasion‐related proteins, such as GATA3, ERK, P21 and E‐ca (Figure 5). There was no significant difference between the overexpression and knockdown experiments.

Overall, miR‐944 inhibits CRC cell proliferation, migration and invasion by interacting with GATA6 and its downstream proteins.

### 3.5 GATA6 knockdown reversed the impacts of silencing miR‐944 in both cell lines

**Figure 6:**
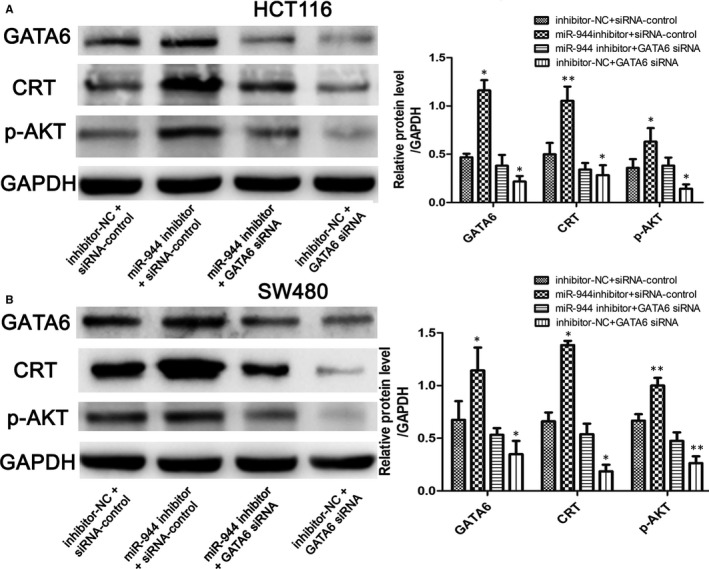
GATA binding protein 6 (GATA6) knockdown reverses the increased expression levels of GATA6, CRT and p‐AKT induced by the miR‐944 inhibitor. A, GATA6 antisense experiments in Human colon cancer cells‐116(HCT116) cells, and (B) GATA6 antisense experiments in SW480 cells, n = 3, *P < 0.05 and **P < 0.01 by *t* test

**Figure 7:**
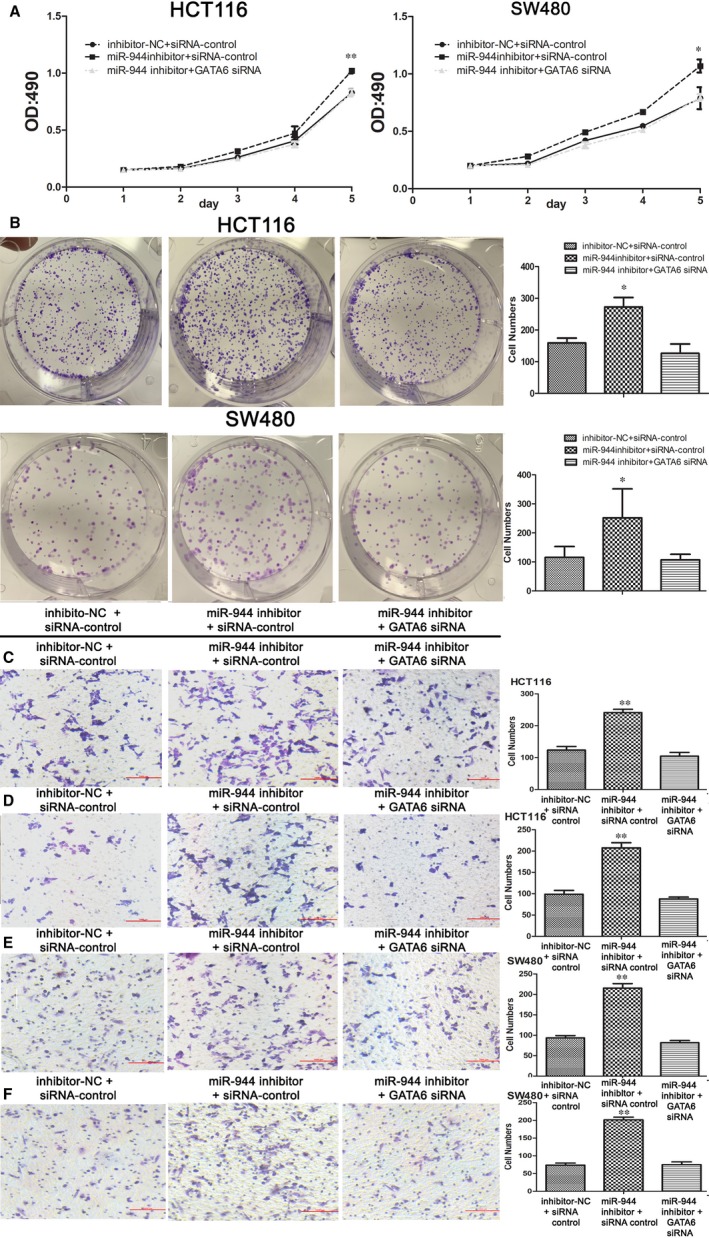
GATA binding protein 6 (GATA6) knockdown reverses cell functions impacted by the silencing miR‐944. A, 3‐(4,5‐dimethyl‐2‐thiazolyl)‐2,5‐diphenyl‐2‐H‐tetrazolium bromide, Thiazolyl Blue Tetrazolium Bromide (MTT) assays of Human colon cancer cells‐116(HCT116) and SW480 cells are among the three groups described. B, Colony formation assays of HCT116 and SW480 cells. (C,E) Cell migration assays of HCT116 and SW480 cells. (D,F) Cell invasion assay of HCT116 and SW480 cells

To verify the relationship between miR‐944 and GATA/CRT/p‐AKT in CRC, we performed an anti‐sense experiment. First, we found that the expression level of GATA6 was significantly reduced when transfecting GATA6 siRNA. GATA6 knockdown significantly reversed the increased expression levels of the CRT and p‐AKT proteins induced by the miR‐944 inhibitor compared with the inhibitor‐NC and siRNA‐control groups in HCT116 and SW480 cells (Figure 6). To confirm that miR‐944 affects CRC cell function by GATA6, we investigated the proliferation, migration and invasion of HCT116 and SW480 cells after interfering with the expression of miR‐944 and GATA6. The MTT and colony formation assays showed that transfecting GATA6 siRNA reversed the increased CRC cell proliferation induced by miR‐944 inhibitor (Figure 7A,B). Furthermore, the cell migration and invasion assays showed that GATA6 knockdown reversed cell migration and invasion (Figure 7C‐F).

## 4 DISCUSSION

Has‐miR‐944 is a conserved non‐coding RNA sequence. Previous studies have shown that miR‐944 plays the opposite role in different human tumours. In cervical cancer and endometrial cancer,^18^, ^19^ the expression of miR‐944 is significantly upregulated. However, several studies have demonstrated that a high expression level of miR‐944 is associated with better prognosis in human cancers, such as gastric cancer, bladder cancer and non‐small cell lung cancer.^14^, ^20^, ^21^ In this study, we analysed the expression of miR‐944 in 100 pairs of human CRC tissues and adjacent tissues and four CRC cell lines by qRT‐PCR. The results showed that miR‐944 expression was significantly downregulated, and HCT‐116 cells had the lowest miR‐944 expression level and SW480 cells had the highest miR‐944 expression level. Moreover, the clinicopathological data showed that a high expression level of miR‐944 is negatively associated with the TNM stage, depth of invasion and lymph node status. Tumour cell proliferation, invasion and migration are important factors affecting CRC patient survival. Therefore, our subsequent experiments showed that the restoration of miR‐944 expression in CRC cells inhibits cell proliferation, invasion and migration, indicating that miR‐944 is likely a novel target for CRC therapy.

Our subsequent experiments showed that GATA6 is the target of miR‐944 that was not reported previously to our knowledge. In the 40 CRC tissues, there was a negative association between miR‐944 expression and GATA6 expression. GATA transcription factors are a series of zinc finger proteins that can determine the consensus DNA sequence WGATAA.^22^ The GATA family consists of six members (GATA1‐6),^23^ and GATA6 is located on 18q11.2 and participates in cell differentiation of the splanchnic mesoderm, such as the lung and gastrointestinal track.^24^ Emerging evidence has shown that GATA6 acts as a tumour promoter in CRC. Hironori Ushijima et  al^25^ showed that the degradation of GATA6 in CRC cell lines inhibits cell proliferation at the progression of the G2/M phase, and cells are more sensitive to chemotherapy by likely regulating JNK signalling. Dysregulation of GATA6 expression has been shown to be significantly associated with liver metastasis (*P* = 0.001) and poor overall survival (OS) (*P* < 0.001), and knockdown of GATA6 has been shown to suppress cancer cell invasion.^16^ However, the mechanism by which overexpression of GATA6 inhibits the initiation and progression of CRC is not known. Our subsequent study aimed to determine the potential target of GATA6.

GATA6 has been experimentally identified as the transcription factor that binds to the promoter of calreticulin (CRT) by reporter gene and ChiP analyses in mice and humans.^26^ However, no reports were definitely verified the relationship between GATA6 and CRT in cancers to our knowledge. In this study, we also found that the CRT protein changes with the level of GATA6 protein expression in CRC cell lines. Therefore, we concluded that miR‐944 mediates CRT protein expression by inhibiting GATA6 protein expression. In recent years, CRT expression has been reported to be associated with more invasive and advanced malignant processes as well as a poorer prognosis and is positively associated with the UICC stage and lymph node metastasis in several cancers,^27^, ^28^ such as breast cancer and pancreatic cancer. Several pathways have been reported to be related to malignant tumour function, such as the modulation of Slug/E‐cadherin,^31^ PI3K‐Akt^32^ and MEK‐ERK.^33^ Because the PI3k‐AKT pathways were activated and upregulated by CRT and the expression of E‐cadherin and ERK was not impacted by miR‐944 in this study, we performed an immunoprecipitation experiment in HCT116 and SW480 cells and found that CRT is structurally associated with AKT. However, the Western blot results showed that miR‐944 overexpression or downregulation of miR‐944 expression can affect p‐AKT protein expression without changing the expression levels of total AKT. The PI3K/AKT signalling pathway participates in the progression of cell proliferation, migration and apoptosis in CRC,^34^ and it may be the underlying mechanism by which miR‐944 affects CRC cell functions. In fact, the mechanism of miR‐944 in CRC is complicated. miR‐944 may affect other functions of CRC cells, and perhaps there are many cross‐interactions of factors and signalling pathways that need to be further investigated.

In conclusion, our present study provided evidence that miR‐944 acts as a tumour suppressor that is significantly downregulated in CRC tissues. For the first time, we showed that miR‐944 inhibits CRC cell proliferation, invasion and migration by regulating GATA6 and its downstream proteins. Moreover, the expression of miR‐944 was shown to be significantly associated with the TNM stage, depth of invasion, and lymph node status. The interaction between miR‐944 and the GATA6 pathway might provide new insight for demonstrating malignant biological behaviour and providing new gene‐targeted therapies for CRC.

## CONFLICT OF INTEREST

The authors declare no conflict of interest.
