# Supplementary material for: Ectopic expression of miR‐944 impairs colorectal cancer cell proliferation and invasion by targeting GATA binding protein 6
Source: J Cell Mol Med. 2019 Mar 15;23(5):3483–94. doi: 10.1111/jcmm.14245 (PMC6484418; doi:10.1111/jcmm.14245)
Supplement: Supplementary file 1 [file JCMM-23-3483-s001.docx]

**Table S1**. miR-944 expression of 40 pairs’ CRC and its corresponding Immunohistochemistry (IHC) scoring.

| NO. of CRC | delt CT | IHC scoring |
| --- | --- | --- |
| 315 | 13.47 | 12 |
| 316 | 13.8 | 9 |
| 318 | 2.73 | 1 |
| 320 | 12.57 | 6 |
| 321 | 6.68 | 2 |
| 324 | 8.67 | 0 |
| 325 | 12.45 | 12 |
| 327 | 10.62 | 8 |
| 328 | 13.8 | 9 |
| 329 | 10.86 | 8 |
| 330 | 7.1 | 6 |
| 331 | 14.86 | 8 |
| 333 | 8.06 | 8 |
| 334 | 7.78 | 4 |
| 335 | 13.43 | 8 |
| 336 | 7.58 | 4 |
| 337 | 9.32 | 1 |
| 339 | 5.55 | 3 |
| 340 | 12.54 | 12 |
| 341 | 6.95 | 3 |
| 343 | 8.83 | 0 |
| 344 | 10.65 | 4 |
| 345 | 8.97 | 6 |
| 346 | 8.03 | 8 |
| 347 | 6.89 | 3 |
| 348 | 9.79 | 4 |
| 349 | 12.77 | 0 |
| 350 | 10.54 | 12 |
| 351 | 12.77 | 8 |
| 352 | 13.94 | 12 |
| 353 | 12.93 | 12 |
| 354 | 2.36 | 4 |
| 357 | 0.77 | 0 |
| 358 | 9.82 | 8 |
| 360 | 10.29 | 3 |
| 361 | 8.2 | 0 |
| 362 | 9.56 | 8 |
| 363 | 12.35 | 4 |
| 364 | 9.36 | 12 |
| 365 | 14.76 | 4 |

we identified that delt CT≥9 is miR-944 low expression and IHC scoring ≥8 is miR-944 high expression.
